# Ovarian stimulation with GnRH analogues

**DOI:** 10.5935/1518-0557.20140003

**Published:** 2014

**Authors:** Ivan S. Montenegro, Mariana Faller, Isabel C. A. de Almeida, Eduardo P. Passos

**Affiliations:** 1 Gynecology and Obstetrics Service of the Hospital de Clínicas de Porto Alegre, Assisted Reproductive Sector, Federal University of Rio Grande do Sul; 2 SEGIR- Serviço de Ecografia, Genética e Reprodução Humana

**Keywords:** Assisted reproductive technology, equity, access, social class, public health care

## Abstract

**Objective:**

Analysis and compare data between two induction protocols (long agonist and flexible antagonist) in patients submitted an assisted reproduction technique in Porto Alegre.

**Methods:**

Cross-sectional study comparing the intermediate results with the use of two different ovarian stimulation protocols with gonadotropin-releasing hormone agonist versus antagonist to assisted reproductive techniques. The statistical analysis of the retrieved data (age, body mass index, number of oocytes recovered, number of fertilized oocytes, number of oocytes cleaved, total dose of FSH used and ovarian hyperstimulation syndrome) was performed by Student’s t-test for parametric data and analysis of covariance for the dependent variables.

**Results:**

A total of 50 patients, 25 in each group, met the criteria for inclusion in the study between January and March in the year 2010. There was statistically significant difference only in the middle ages between the groups (P = 0.031). There was no statistical difference for the remaining data analyzed (body mass index, number of oocytes recovered, number of fertilized oocytes, number of oocytes cleaved and dose of FSH utilized). There were no cases of ovarian hyperstimulation syndrome.

**Conclusion:**

Both protocols are equal in terms of results. The agonist has advantages about scheduling of the procedure, but it takes too long to start the stimulation and have possibility to start medication in a pregnant patient. Added to this, we have the possibility of getting the ovarian hyperstimulation syndrome as complication. In the antagonist group, is clear the ease-of-use of the medication and the fastest start of stimulation.

## INTRODUCTION

The reproductive treatment for infertile couples has evolved rapidly since the birth of Louise Brown, the first baby born from in-vitro fertilization (IVF), performed by Steptoe and Edwards in 1978 (Steptoe & Edwards, 1978). New treatment techniques, new technologies used in the laboratory and new medications make up an arsenal of options in these cases and have helped in the treatment of these couples. The new medications have brought a considerable increase in the chance of pregnancy at the time controlled ovarian stimulation (COS) allowed an increase in the number of oocytes to be recruited and, consequently, greater number of embryos (Yovich, 2011).

The gonadotropin-releasing hormone (GnRH) analogues (agonist and antagonist), in association with exogenous gonadotropins brought the solution to one of the COS’s problems: the early peak of luteinizing hormone (LH), which harmed the oocytes collection around 20% of the cases (Neveu *et al.*, 1987; Ashkenazi *et al.*, 1989). The GnRH agonists’ analogues act occupying the GnRH receptors in the pituitary gland causing desensitization. Initially, this medication releases ever follicle-stimulating hormone (FSH) produced and stored in granules, to then, block the production of the pituitary hormones (FSH and LH) (Magon, 2011). The initial pituitary lock occurs between 3 and 4 weeks after the start of the use of the medication and its effect is long-lasting, and may persist for a few weeks after the discontinuation of use (Magon, 2011). Such use prevents the early release of LH, enabling greater control of the stimulus and it is most used in the middle of the luteal phase of the previous cycle (long protocol) (Madani *et al.*, 2012). The advent of antagonist brought another perspective of handling because its use happens to be held at COS cycle itself. The antagonists act in the pituitary gland through a competitive blocking at GnRH receptors and its action is dose dependent. It occupies the receptors without stimulating the secretion of gonadotropins and the final effect is not to release of pituitary hormones, especially the LH. The onset of action is immediate and the duration of the effect lasts around 34 hours (Schally, 1999; Al-Inany *et al.*, 2011; Gilliam, 2011).

The most recent studies result that two induction protocols are equivalent in terms of retrieved oocytes, embryos fertilized, pregnancy and birth rates. However, they reveal that the protocol that uses antagonist appears to be safer due to the lower occurrence of ovarian hyperstimulation syndrome (OHSS), and it ensures that the patient is not pregnant when the beginning of the use of medicines. Still, the literature has shown a greater need for gonadotropins use with the agonist protocol, resulting in higher cost for patients in addition to the risk of OHSS. (Del Gadillo *et al.*, 2002; Prapas *et al.*, 2005; Aletebi, 2007; Al-Inany *et al.*, 2011; Bodri *et al.*, 2011; Gilliam, 2011).

In our service routinely has been used the agonist protocol, but the possibility of antagonists usage results in greater ease of handling by the user, involves a single menstrual cycle and dispensation guidance for drug use at the end of the previous cycle. Thus, we use the protocol with antagonists and, in this way, we seek to evaluate the use of the two protocols and analyze their results. Added to this, the fact that there is no study that make this comparison in our population.

## MATERIAL AND METHODS

The study constitutes a cross-sectional cohort comparing the intermediate results in assisted reproduction between the GnRH agonist long protocol (group 1) and flexible GnRH antagonist protocol (group 2), in patients undergoing IVF and intracytoplasmic sperm injection (ICSI) procedure in the year 2010.

For patients in group 1 were administered GnRH agonist, nafarelin acetate 200 µg/day (Synarel^®^; Pfizer, USA), intranasal spray, beginning in the 21st day of the cycle prior to stimulation. The administration of recombinant FSH, follitropin β 150 UI/day (rFSH, Puregon®; Organon, The Netherlands), s.c, began on day 3 of the cycle. The dose of GnRH agonist was decreased on that day to 100 µg/day and continued until and including the day of triggering of final oocyte maturation.

In the group 2, patients started daily rFSH treatment with s.c injections of 150 UI of follitropin β (Puregon®, Organon, The Netherlands), on day 3 of cycle. Daily s.c administration of ganirelix 0.25 mg (Orgalutran®, Organon, The Netherlands) was initiated when at least one follicle measuring >14 mm is the ultrasound scanning. Treatment with rFSH and GnRH antagonist continued daily thereafter, until and including the day of triggering of final oocyte maturation.

The dose of rFSH was fixed in both groups, and, in the 5th day of stimulation, the gonadotrophin was changed to highly purified human menopausal gonadotrophin, menotropin 150 UI/day (HMG, Menopur®, Ferring, Germany), s.c. As soon as three follicles reached a mean diameter of ≥17 mm on ultrasound scanning, recombinant human chorionic gonadotrophin, choriogonadotropin alfa 250 µg, (rHCG, Ovidrel®, Serono, Switzerland) were administered s.c.

Oocyte retrieval was performed 35-36 h after the hCG injection by transvaginal ultrasound-guided needle aspiration. ICSI was performed only in cases with severe male factor or previous fertilization failure. Ultrasound guidance was used for all embryo transfers, which were performed 2 or 3 days post-oocyte retrieval, depending on the weekly clinical program (i.e. to avoid embryo transfer on Sundays). Luteal phase support with 600 mg of micronized progesterone (Utrogestan®. Besins-International S.A., France) was initiated in the same day of oocyte retrieval. Were excluded patients with indication of ICSI of testicular biopsy and included patients who failed in low complexity treatments, like intrauterine insemination (IUI). The data were extracted from electronic medical records, being analyzed: date of birth, date of the procedure (ovarian puncture), COS protocol, body mass index (BMI), number of oocytes retrieved, number of fertilized oocytes, number of oocytes cleaved, units of FSH (uFSH) necessary to proceed COS and occurrence of OHSS.

Whereas a difference of up to 3 oocytes as equal among the groups studied, and a standard deviation of 2.7 and 3.2 respectively and each group according to found in the previous study (Gilliam, 2011) were needed at least 34 fertilization cycles, being 17 in each group. Then, for statistical analysis, we used the Student’s t-test, Analysis of Covariance (ANCOVA) or Chi-Square test when appropriate, calculated with the program SPSS 16.0. The work was submitted and approved by the Hospital de Clínicas de Porto Alegre’s committee of ethics in Research.

## RESULTS

We selected the first fifty patients’ files who underwent do IVF/ICSI procedures in 2010 (between January and March), being 25 in each group. The average age of the Group 1 was 36.88 years, statistically greater than group 2, which was 33.96 (P < 0.05) (table 1). There was no difference between the averages BMI between the groups. Fixing up by age, with the ANCOVA, the comparison of averages oocytes recovered, fertilized oocytes, embryos cleaved and uFSH necessary to proceed COS (table 2). There were no cases of OHSS in both groups.

**Table 1:** Patient demographic data for the GnRH-antagonist and GnRH-agonist groups

Included Pacients (n = 50)	Group 1 Agonist Long Protocol (n = 25)	Group 2 Antagonist Flexible Protocol (n = 25)	*P*†
Age (years)	33.96 ± 5.00	36.88 ± 4.26	.031
BMI* (kg/m2)	23.05 ± 2.96	24.70 ± 2.80	.722

Note: Data listed as mean ± standard derivation (SD)

* Body Mass Index †Student’s t-test

**Table 2:** Assisted reproduction outcomes

Included Pacients (n = 50)	Group 1 Agonist Long Protocol (n = 25)	Group 2 Antagonist Flexible Protocol (n = 25)	*P*
Oocyte Retrieval	5.39 ± 0.976	6.89 ± 0.976	.288†
Fertilized Oocytes	3.28 ± 0.551	3.08 ± 0.551	.803†
Cleaved Embryos	3.00 ± 0.449	2.56 ± 0.449	.549†
uFSH*	1449.77 ± 33.704	1432.23 ± 33.704	.719†

Note: Data listed as mean ± Standard error (SE) corrected by age with ANCOVA

* units of FSH †ANCOVA

## DISCUSSION

As already established in the literature, both protocols are similar (Loutradis *et al.*, 2004; Prapas *et al.*, 2005; Xavier *et al.*, 2005; Aletebi, 2007; Kurzawa *et al.*, 2008; Hosseini *et al.*, 2010; Tehraninejad *et al.*, 2010; Al-Inany *et al.*, 2011; Bodri *et al.*, 2011; Gilliam, 2011; Pu *et al.*, 2011; Haydardedeoglu *et al.*, 2012; Pundir *et al.*, 2012). We found the same results in our study.

As demonstrated in this study, there is no difference in oocytes retrieval between these two protocols in recent meta analysis (Tehraninejad *et al.*, 2010; Bodri *et al.*, 2011; Pu *et al.*, 2011; Casano *et al.*, 2012; Pundir *et al.*, 2012) and randomized controlled trials (Loutradis *et al.*, 2004; Xavier *et al.*, 2005; Kurzawa *et al.*, 2008; Tazegul *et al.*, 2008). Differently, some reports showed a less number of oocyte retrieval in the antagonist protocol (Barmat *et al.*, 2005; Arruda *et al.*, 2013; Orvieto & Patrizio, 2013). Our results could be discussed in another way when we decide to use minor dose of gonadotropin. We could say: minor dose, less oocyte, less complication. We are getting more reliable lab results so that we do not want much cryopreserved embryos. In this way we think that a friendly stimulation with a sufficient oocyte to have embryo to be transferred and a little to be cryopreserved should be routine, and, in a future, it should be fertilized only a minimal necessary oocyte and cryopreserve oocyte. Our results demonstrate a more number of oocyte in the antagonist protocol with no significant difference between both groups.

We found no difference in the fertilized oocyte in both groups. We do know the quality of the oocyte is import for the lab results (Xia, 1997) so that in both protocols our operator demonstrates an accuracy of decision in terms of puncture day. Concerning the lab, we could say that we advanced in the last years so that even with some immature we could find good results (Krisher, 2004; Borini & Bianchi, 2012). It is interesting that all lab results are almost equal in terms of percentage of fertilization and cleavage. So, we could say it depends on the operator experience in the use of those protocols. We do know the more antagonists, the less we get LH so that we should think in a mean LH level in terms of results. Recently, Papanikolaou *et al.* published a correlation between progesterone levels and pregnancy rates with no difference between protocols but an interesting data in terms of progesterone level, that we got no results because had no routine relating to this data (Papanikolaou *et al.*, 2012).

The antagonist protocol is protective concerning OHSS because even with this probably situation we could trigger with agonist and prevent this important complication (Papanikolaou *et al.*, 2011; Humaidan *et al.*, 2012; Lavorato *et al.*, 2012). This fact is of utmost importance specially if we are leading with younger patients. The major age of the patients was a primary indication for the use of antagonist protocol. Anyway we found this aspect of no importance because our data showed the same number of oocyte collected and no cases of OHSS, probably because our sample was not calculated to find difference in OHSS cases or because we had just one case of polycystic ovary syndrome among the cases studied. We also believe that the minor dose of gonadotropins used in our protocols is responsible for the success in avoid OHSS in both protocols, especially in the agonist protocol.

The agonist is more comfortable for the organization of the oocyte retrieval day but it should be used on the previous cycle and could get patients that might be pregnant (Hayden, 2008). In our study we found no problem about this fact because of the routine imposed but it should be discussed with the group about the facility of some flexible puncture date. Tremellen and Lane showed in an article demonstrating that we could use more antagonist to postpone the puncture day with no adverse results (Tremellen & Lane, 2010).

After corrected by age, our study shows a minor dose of uFSH in antagonist protocol, but with no significant difference when compared to agonist protocol. Some reports evidence that a less amount of uFSH is needed in antagonist protocol (Kurzawaa *et al.*, 2008; Tazegul *et al.*, 2008; Lainas *et al.*, 2010; Tehraninejad *et al.*, 2010; Pundir *et al.*, 2012; Arruda *et al.*, 2013) and other papers shows no difference between protocols (Barmat *et al.*, 2005; Bodri *et al.*, 2011). The small need of uFSH determine a more friendly stimulation with less risk of OHSS and a greater compliance of our patients in treatment.

## CONCLUSIONS

Finally we should say both protocols are equal in terms of results (Depalo *et al.*, 2012; Luna *et al.*, 2012). The agonist has advantages concerning the mobility but it get to long to start the stimulation and, even, could get a pregnant patient and adjunctive the possibility of getting the OHSS as complication (Zorn *et al.*, 1987; Depalo *et al.*, 2012). In the antagonist group, it is clear the facility of usage and the faster stimulation cycle as it start in the same puncture cycle (Depalo *et al.*, 2012). The idea of impossibility of mobility was not a compliance of our group and should be discussed the possibility of use of more antagonist in case of discomfort of schedule but implicate in a more costs in protocol. It is expected that the availability of evidence-based information on the optimal use of GnRH antagonists will enhance the probability of pregnancy associated with their application in ovarian stimulation (Xavier *et al.*, 2005). In addition to the flexible antagonist protocol be safer in terms of less likelihood of OHSS and the patient could not be pregnant at the beginning of the cycle (Al-Inany *et al.*, 2011; Depalo *et al.*, 2012), we also consider that the fact of the shorter duration of the stimulus and the least amount of FSH units required are economic factors that also influence the therapeutic decision of the human reproduction team. The smallest amount of gonadotropins directly decreases the costs of treatment, since, in this case, you will need to acquire the least amount of medication to the induction cycle. Already the smallest stimulus duration decreases treatment costs indirectly, as decrease transportation costs to the place of treatment, the shortest time of absence at work and other indirect expenses that are difficult to calculate.
